# Association Between Changes in Sleep, Nap Duration and Bone Mineral Density in Mexican Adults

**DOI:** 10.1007/s00223-024-01224-1

**Published:** 2024-05-17

**Authors:** Joacim Meneses-León, Sonia Hernández-Salazar, Karina Robles-Rivera, Marcela Tamayo-Ortiz, Karla Muciño-Sandoval, Rodolfo Rivas-Ruiz, Edgar Denova-Gutiérrez, Juan A. Tamayo-Orozco, Rafael Velázquez-Cruz, Jorge Salmerón, Berenice Rivera-Paredez

**Affiliations:** 1 Research Center in Policies, Population and Health, School of Medicine, National Autonomous University of Mexico (UNAM), Zona Cultural S/N, CIPPS 2° Piso Ciudad Universitaria, Coyoacán, C.P., 04510 Mexico City, Mexico; 2 Secretary of Clinical Teaching, Medical Internship, and Social Service, School of Medicine, UNAM. Circuito Interior, Ciudad Universitaria, Avenida Universidad 3000, C.P., 04510 Mexico City, Mexico; 3 Department of Environmental Health Sciences, Mailman School of Public Health, Columbia University, 722 West 168th Street, New York, NY 10032 USA; 4 Accessalud, Av. Insurgentes Sur 299, Hipódromo, Cuauhtémoc, C.P., 06100 Mexico City, México; 5 Training and Clinical Research Center, Health Research Coordination. Mexican Social Security Institute (IMSS), Av. Cuauhtémoc #330, Col. Doctores, C.P., 06720 Mexico City, Mexico; 6 Center for Research in Nutrition and Health, National Institute of Public Health (INSP), Mexico. Av. Universidad #655, Col. Santa María Ahuacatitlán. C.P., 62100 Cuernavaca, Morelos, Mexico; 7 Genomics of Bone Metabolism Laboratory, National Institute of Genomic Medicine (INMEGEN), Periférico Sur No. 4809, Col. Arenal Tepepan, Alcaldía Tlalpan, C.P., 14610, Mexico City, Mexico

**Keywords:** Sleep duration, Nap, Bone mineral density, Mexican population

## Abstract

Studies have found associations between sleep, nap duration, and bone mineral density (BMD). However, the longitudinal relationship between sleep, nap duration, and BMD has not been explored. We evaluated the association between the change in sleep and nap duration and BMD in Mexican adults. Data come from 1,337 adult participants of the Health Workers Cohort Study (341 were men and 996 were women, including 450 women < 45 years old and 546 ≥ 45 years old), with two study waves. At each wave, sleep and nap duration was assessed using self-administered questionnaires and BMD in g/cm^2^ was determined by dual X-ray absorptiometry. We used fixed-effect regression models stratified by sex and adjusted for BMI, diet, physical activity, vitamin supplements, and hormone replacement therapy. Women who changed from < 7 to ≥ 7 h/day of sleep from baseline to follow-up were associated with increases in the total hip (β = 0.012 g/cm^2^; 95% CI: 0.002, 0.022) and lumbar spine BMD (β = 0.024 g/cm^2^; 95% CI: 0.009, 0.039). Furthermore, most of these associations were observed in women ≥ 45 years. For women, a changing from 0 to > 60 min/day of napping was associated with a significant increase in total hip BMD of 0.012 g/cm^2^ (95% CI: 0.004, 0.024) and lumbar spine BMD of 0.027 g/cm^2^ (95% CI: 0.009, 0.045). No significant associations were observed for men. Our results suggest that increased sleep and nap duration are associated with gains in BMD in Mexican women, emphasizing sleep’s role in promoting bone health and supporting established recommendations.

**Supplementary Information:**

The online version contains supplementary material available at 10.1007/s00223-024-01224-1.

## Introduction

Sleep, a vital biological process, influences various metabolic and endocrine functions[1]. Poor sleep is associated with health conditions, including obesity, diabetes, hypertension, cardiovascular disease, and mortality[2–4]. Evidence suggests that sleep and naps also impact processes related to body composition, including bone health[5–8]. Osteoporosis, characterized by reduced bone mineral density (BMD), significantly raises the risk of fractures, posing a global public health concern[9, 10]. Statistics indicate that about one in three women and one in five men over 50 worldwide will experience a BMD-related fracture[11]. Additionally, recent findings have highlighted a consistently increased risk of post-hip fracture mortality with low socioeconomic status (SES) across various measures of SES and different countries with diverse political structures and health and social care infrastructures[12]. A recent study in Mexico has revealed a decreasing trend in hip fracture rates since 2006, particularly among individuals aged 60 and older [13]. Rates declined from 167.8/100,000 in 2006 to 138.5/100,000 in those aged 60 and over, with annual declines of 1.9% in women and 0.9% in men. Older age cohorts, notably those born before 1937, accounted for the majority of hip fractures. However, as the population ages in the coming decades, these declines may be offset by an increasing proportion of older individuals, potentially reversing these trends. These findings emphasize the importance of age, sex, and geographic considerations in interpreting bone health outcomes in Mexico [13].

Previous cross-sectional studies found associations between inadequate or excessive sleep (beyond 7 to 9 h per night) and lower BMD or osteoporosis[14–17]. For instance, older adults women with more than 3.4 h of weekly napping have a lower BMD[7]. Recent studies also link poor sleep patterns to an increased risk of osteoporosis[18, 19]. While sleep disturbances have been associated with a higher fall risk and increased fracture risk[20], their direct impact on BMD remains unclear. Few studies have specifically investigated how changes in sleep and nap duration relate to bone health, particularly BMD. Understanding this connection is crucial for informing preventive strategies and interventions against osteoporosis and fractures.

The biological mechanism explaining the effects of sleep and nap duration on BMD remains uncertain. However, reduced BMD in individuals with restricted sleep may result from disruptions in circadian rhythm-regulating clock genes, leading to alterations in bone turnover markers, increased endogenous glucocorticoid secretion, and growth hormone inhibition[6, 7, 18, 21].

The current increase in life expectancy and longevity implies greater risks of osteoporosis and related fractures, and therefore a major economic burden for Mexico[10]. In recent years, there has been an increase in the prevalence of short sleep duration in Mexico; according to the Mexican National Health and Nutrition Survey (ENSANUT, by its Spanish acronym) with 28.4 and 30.7% of adults reporting sleeping 7 h or less per day in 2016 and 2022, respectively[22, 23]. Given the frequency of short sleep and its impact on BMD, it is imperative to investigate the relationship between sleep duration, napping, and bone health in the Mexican population. Furthermore, hormonal changes, particularly in adults aged 45 years and older, may play a significant role in mediating the relationship between sleep patterns and bone health. Hormones such as estrogen, which decline with age, have well-established effects on bone metabolism and mineralization[24, 25]. Reduced levels of estrogen in postmenopausal women, for example, are associated with accelerated bone loss and increased risk of osteoporosis and fractures[25, 26]. Therefore, our study aims to evaluate the association between changes in sleep and nap duration and BMD in Mexican adults, recognizing the need for context-specific insights into this intricate interplay within our society.

## Methods

### Study Population

We used data from the Health Worker Cohort Study (HWCS) for this longitudinal analysis. The HWCS is an open prospective cohort to examine the association between lifestyle and genetic factors and chronic disease. Participants were recruited through leaflets distributed at the Mexican Social Security Institute (IMSS, by its Spanish acronym) from 2004 to 2006 (Wave 1) and followed up in 2010–2012 (Wave 2). Details of the study design and methods have been published previously[27].

The inclusion criteria for the present analysis were defined as adult men and non-pregnant women (≥ 20 years old) not exposed to radiation three months before enrollment in the study and without implanted defibrillator devices or prostheses. We excluded participants with missing data for BMD measurement (*n* = 302), sleep duration (*n* = 185), physical activity (*n* = 3), smoking status (*n* = 35), or diet (*n* = 25), resulting in a final sample of 1,337 individuals with complete information regarding the study variables in the two waves.

## Sleep Duration

Sleep duration was calculated using the answer to the question “How long do you sleep per day?” and categorized as < 7 and ≥ 7 h. Short sleep duration was determined as sleeping less than 7 h per day, considering that the recommended sleep duration for adults was about 7–9 h per day[28]. Additionally, other categories of sleep duration, such as (1–4, 5–6, 7–8, and ≥ 9 h/day)[29], were also evaluated to provide a comprehensive assessment of the relationship between sleep duration and bone health. Nap duration was calculated using the answer to the question “How many minutes do you usually nap during the day?”. Sleep and nap duration separately asked for weekdays and weekends. Participants were asked to report the average duration of their naps on a 7-point scale: < 15, 16–29, 30–59, 1−2, 3–4, 5–6, and > 6 h. If participants did not provide a response to this question, nap duration was recorded as 0 min, as per the design of the questionnaire. This decision was made due to the absence of a specific option for indicating ‘I do not engage in this activity’. We used the average for each category weekday/weekend. Total nap duration was classified into four categories: 0, 0–30, 30–60, and > 60 min[18]. This classification approach aligns with that employed in a previous study by Cheng et al., which investigated similar associations. Ensuring methodological consistency facilitates comparability of results across different populations.

## Bone Mineral Density

Dual-energy X-ray absorptiometry (DXA) (GE-lunar Prodigy, WI, USA) whole body scans were used to measure subtotal (including spine, ribs, pelvis, and extremities) and regional (total hip, and lumbar spine) BMD as well as percent body fat. DXA was calibrated daily using a standard phantom provided by the manufacturer. Measurements were maintained within the manufacturer’s precision standards[27]. Low-BMD was defined as a T-score below −1 at lumbar spine and total hip following World Health Organization (WHO) criteria[30]. We did not consider the femoral neck measurement due to operator error [31].

## Other Covariates

Participants completed a self-administered questionnaire at both study waves, providing information on birth date, education, medical history, current medication use (including calcium supplements), and lifestyle factors (e.g., diet, smoking, alcohol). In our study, the term sex will be employed to refer to biological distinctions. They also visited our research center for a physical examination and fasting blood sample collection. Dietary intake was assessed with a semi-quantitative FFQ previously validated in a Mexican population[32]. This questionnaire captured data on the frequency of consumption of 116 food items over the past year. Average daily nutrient intakes were calculated by multiplying the frequency of consumption of each food by the nutrient content[27]. We obtained information on nutrient intake from a comprehensive database of food contents[33]. Dietary inflammatory index (DII) score was derived from 30 of the 45 parameters following the methodology proposed by Shivappa et al.[34]. Smoking status was classified into three categories: current, past, and never. Leisure time physical activity (LTPA) was assessed using the Spanish-translated version of the Nurses’ Health Study physical activity questionnaire, validated in Mexican population[35]. The questionnaire estimated weekly LTPA duration in minutes during a typical week in the past year. LTPA was categorized as either inactive (< 150 min/week) or active (≥ 150 min/week of moderate to vigorous physical activity)[36]. Trained personnel collected anthropometric measurements following standardized techniques[27]. BMI status classification was based on WHO criteria[37]. Type 2 diabetes (T2D) was defined as self-reported physician-diagnosed diabetes, use of hypoglycemic medication, or fasting glucose established cut-off points of ≥ 126.0 mg/dL[38].

## Statistical Analysis

Descriptive statistical analyses stratified by sex and wave were performed using measures of central tendency for continuous variables and frequencies for categorical variables. T-test of matched pairs (for continuous variables) and McNemar’s test (for categorical variables) were used to evaluate differences by wave. Cross-sectional associations between sleep and napping duration and BMD (subtotal, hip, and lumbar spine separately) were determined by sex-stratified linear regression models. In addition, for the categorical outcome (low-BMD at different sites) we used logistic regression. To determine longitudinal associations were determined with fixed-effects regression models, which were stratified by sex[39]. These models allowed us to examine how changes in predictor variables, such as sleep duration and nap duration, related to changes in bone mineral density over time within each individual. In these models, we rigorously controlled for relevant confounding variables, such as body mass index, diet, physical activity, vitamin supplements, and hormone replacement therapy. Additionally, stratification by sex enabled us to assess associations separately for men and women, acknowledging potential gender differences in the relationships between sleep, nap, and bone mineral density. Sleep duration was assessed using categories of < 7 and > 7 h, as well as (1–4, 5–6, 7–8, and > 9 h/day), and as a continuous variable. Similarly, nap duration was evaluated using categories of 0, 0–30, 30–60, and > 60 min, as well as a continuous variable. These models were adjusted for BMI, smoking status (never, former smoker), DII, LTPA, calcium supplements, calcium intake, T2D, and hormone replacement therapy. For both analytical approaches, we stratified the analysis for women by age at baseline (< 45 y ≥ 45 years old) as a proxy for postmenopausal status[40]. We used STATA software version 14.0 (Stata Corp LP, College Station, Texas, USA) for the statistical analyses. All statistical tests were two-tailed, and *p* < 0.05 was considered statistically significant. Adjustments for multiple comparisons were performed to control the risk of type I errors due to conducting multiple statistical tests. The Bonferroni correction method was used to establish the adjusted significance threshold (α/n, *p* = 0.001).

## Results

This analysis included 1,337 participants, 74.5% were women. At baseline, the median age was 46 years (P25-P75: 37–55) and both genders had a median sleep duration of 7.3 h per day (range 6.6–8.0) About 64.0% of men and 71.0% of women met the recommended sleep duration of ≥ 7 h/day. Regarding napping, 73.3% of men and 66.1% of women reported taking naps (Table 1).

**Table 1:** Descriptive characteristics of the study participants at baseline and follow-up

	Males		Females	
	*n* = 341		*n* = 996	
Characteristics	Baseline	Follow-up	Baseline	Follow-up
Age^a^, years	45(36–54)	52(44–61)	46(37–55)	53(44–62)
Body mass index^a^, kg/m^2^	26.5(24.3–28.0)	26.9(24.4–29.5)***	25.7(23.5–28.8)	26.6(23.9–29.4)***
Nutritional status, %				
Overweight, %	48.4	47.8	40.7	41.5
Obesity, %	19.4	20.5	18.2	21.6
Body fat proportion^a^	30.6(27.0–35.0)	32.3(28.9–35.9)***	43.0(38.5–47.1)	44.7(40.9–48.9)***
Leisure time physical activity, %	47.8	41.4	34.6	31.3
Dietary inflammatory index^a^	0.27(−1.17,1.84)	0.54(−0.83,1.94)*	−0.19(−1.51,1.40)	0.38(−1.04,1.83)***
Energy intake^a^, kcal/day	2155(1609–2795)	1914(1437–2436)***	1937(1517–2466)	1690(1258–2199)***
Average weekly sleep duration^a^, hours/day	7.3(6.6–8.0)	7.0(6.3–7.6)*	7.3(6.6–8.0)	7.3(6.3–8.0)***
Weekday sleep duration (hrs/day)	7.0(6.0–8.0)	7.0(6.0–7.0)	7.0 (6.0–8.0)	7.0 (6.0–8.0)
Weekend sleep duration (hrs/day)	8.0(7.0–8.0)	8.0 (7.0–8.0)	8.0 (7.0–9.0)	8.0 (7.0–9.0)
Weekend “Catch-Up” sleep duration (hrs/day)	1.0(0.0–2.0)	1.0 (0.0–1.0)	1.0 (0.0–2.0)	1.0 (0.0–2.0)
Recommended Sleep (≥ 7 h/day), %	64.2	59.2	70.5	63.6*
Very short < 4 h/day,%	2.6	5.0	1.9	5.3***
Short 5–6 h/day,%	33.1	35.8	27.6	31.1
Average 7–8 h/day,%	60.1	55.0	64.8	58.4**
Long ≥ 9 h/day,%	4.1	3.2	5.7	5.1
Napping^a^, min/day	16.1(0–57.5)	11.8(0–38.2)	7.5(0–31.8)	7.5(0–31.8)
Yes, %	73.3	69.5	66.1	66.5
0 min, %	26.7	30.5	33.9	33.5
1− < 30 min,%	35.5	36.7	35.6	37.5
30–60 min,%	13.5	15.8	13.2	13.3
> 60 min, %	24.3	17.0*	17.3	15.8
Sleep duration + napping^a^, hours/day	7.6(6.7–8.5)	7.4(6.6–8.1)**	7.7(7.0–8.4)	7.5(6.7–8.3)***
Diabetes, %	13.5	17.6	10.8	14.6*
Smoking status, %				
Current, %	22.0	15.8	13.6	9.9
Past, %	40.8	51.3*	23.9	29.9*
Calcium intake^a^, mg/day	918(663–1259)	757(522–1085)***	931(690–1333)	757(509–1071)***
Vitamin D^a^, μg/day	4.6(2.9–7.7)	3.4(1.9–5.9)***	5.1(3.4–8.1)	3.6(2.1–6.5)***
Calcium supplements, %	4.7	0.0	17.1	18.0
THR,%	–	–	5.7	4.9
Subtotal body BMD^a^, g/cm^2^	1.124(1.061–1.186)	1.121(1.052–1.189)***	0.986(0.924–1.047)	0.976(0.905–1.042)***
Total hip BMD^a^, g/cm^2^	1.086(1.002–1.179)	1.068(0.977–1.159)***	0.994(0.904–1.087)	0.957(0.869–1.062)***
Lumbar spine BMD^a^, g/cm^2^	1.149(1.040–1.260)	1.153(1.053–1.276)***	1.107(0.999–1.217)	1.077(0.961–1.193)***

^a^Median(P25–P75). Baseline 2004–2006 and follow-up 2010–2012.

*p < 0.05

**p < 0.01

***p < 0.001

**Fig. 1:**
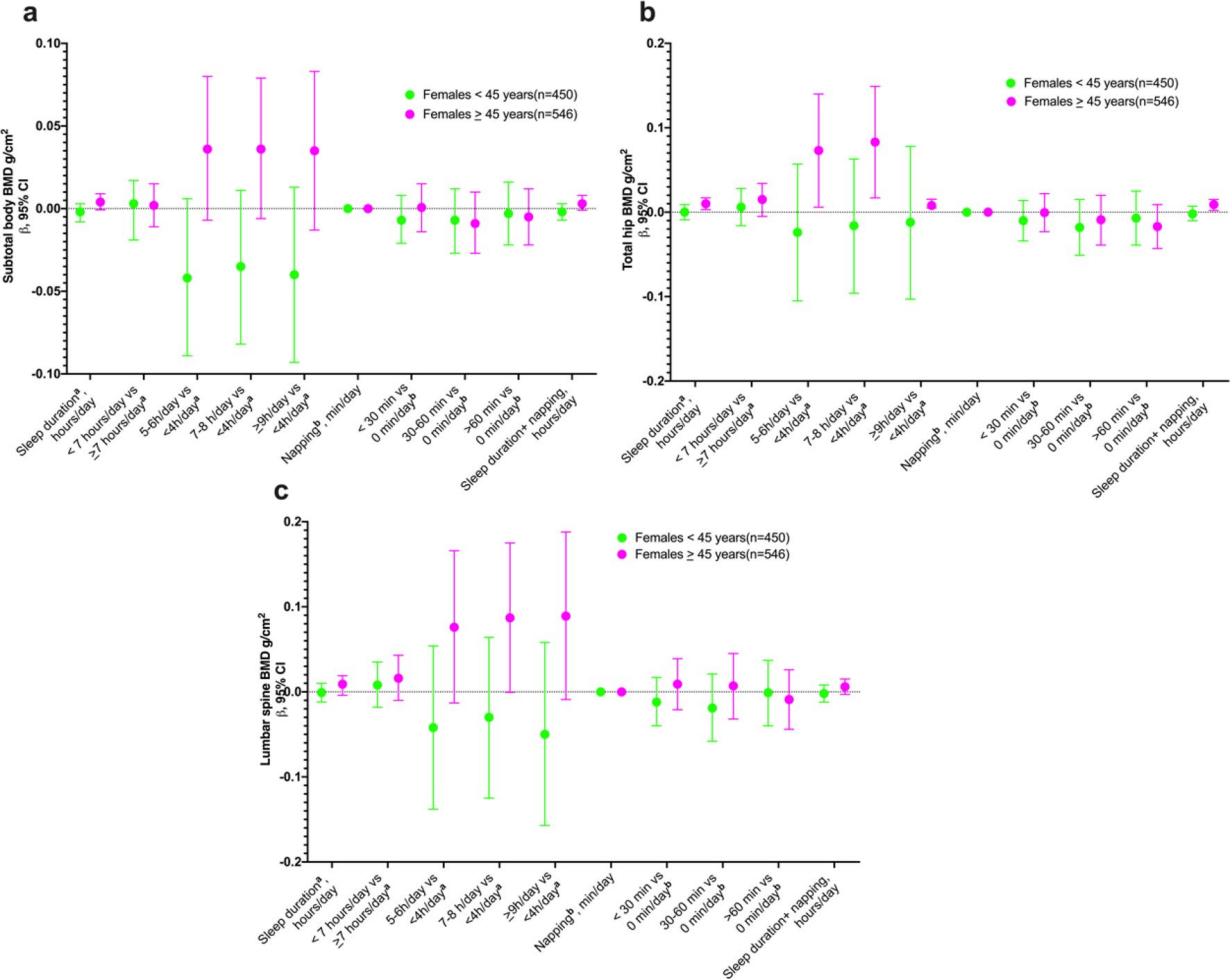
Cross-sectional association of hours of sleep, napping and BMD in women by age groups at multiples sites. **a** Subtotal BMD g/cm^2^, **b** Total hip g/cm^2^, and **c** Lumbar spine g/cm^2^. ^**a**^ This model includes a napping adjustment ^**b**^ This model includes a sleep duration adjustment

**Fig. 2:**
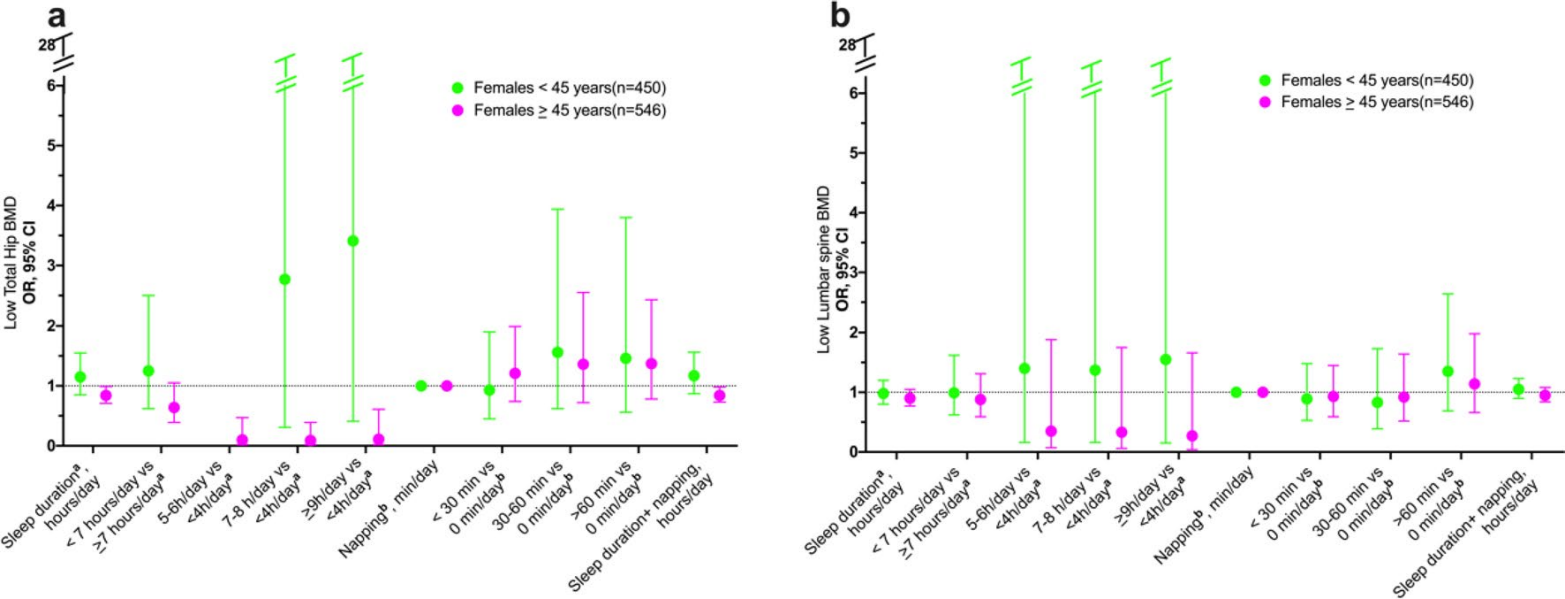
Cross-sectional association of hours of sleep, napping and low-BMD in women by age groups. **a** Low-total hip BMD, and **b** low-lumbar spine BMD. ^**a**^This model includes a napping adjustment ^**b**^This model includes a sleep duration adjustment. Low-BMD as a T-score below −1 at the total hip, and lumbar spine

In the cross-sectional analysis, no sex-specific association was found between sleep duration and BMD. However, among women ≥ 45 years, longer sleep duration (≥ 9 h/day) was positively associated with higher total hip BMD (0.080 g/cm^2^; 95% CI: 0.005, 0.154) compared to those sleeping < 4 h/day. Additionally, there was no observed association between napping and BMD was also observed (Fig. 1 and Supplementary Table 1). Furthermore, a positive association was observed between sleep duration and low total hip BMD in women aged ≥ 45 years, as well as sleep duration plus napping (Fig. 2 and supplementary Table 2).

**Fig. 3:**
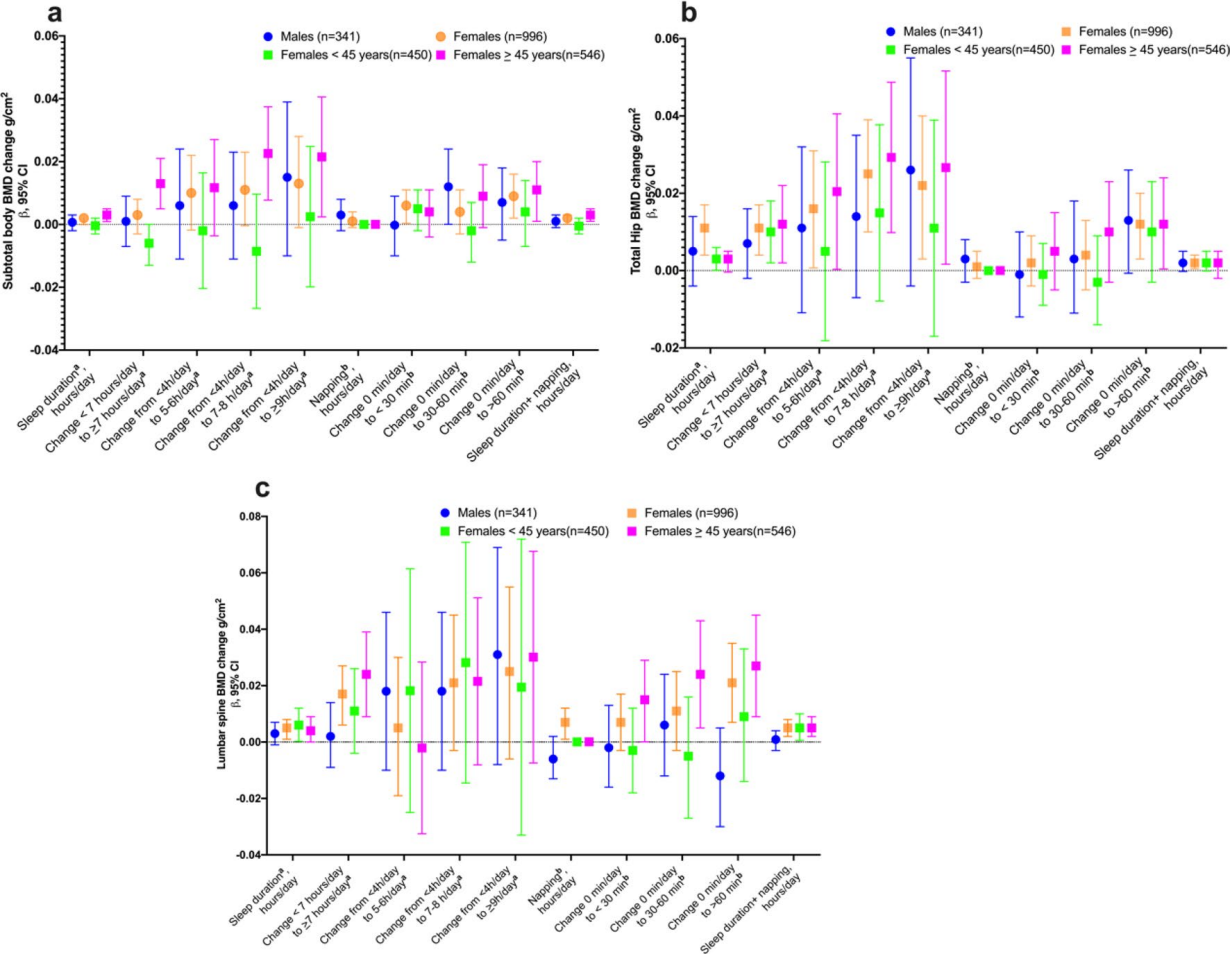
Longitudinal association: BMD change according to Changes in sleep duration between baseline and follow-up by sex and age groups in women at multiples sites. **a** Subtotal BMD g/cm^2^, **b** Total hip g/cm^2^, and **c** Lumbar spine g/cm^2^. ^**a**^ This model includes a napping adjustment ^**b**^ This model includes a sleep duration adjustment

In the longitudinal analysis, we observed an increase in subtotal BMD (0.009 g/cm^2^), total hip BMD (0.012 g/cm^2^), and lumbar spine BMD (0.021 g/cm^2^) in women who changed their nap from 0 min/day to > 60 min/day. In men, we observed an increase in subtotal BMD (0.012 g/cm^2^) for those who changed their nap duration from 0 min/day to 30–60 min/day. However, no significant changes were observed in subtotal, total hip, and lumbar spine regions after adjusting for sleep duration (Fig. 3 and supplementary Table 3).

In the adjusted model stratified by age among women, we observed a subtotal body and lumbar spine BMD gain in women ≥ 45 years who changed from < 7 to ≥ 7 h/day of sleep from baseline to follow-up, with an increase of 0.013 g/cm^2^ (95% CI: 0.005, 0.021) and 0.024 g/cm^2^ (95% CI: 0.009, 0.039), respectively. The total hip BMD gain in women < 45 years and ≥ 45 years who changed from < 7 to ≥ 7 h/day of sleep from baseline to follow-up was 0.010 g/cm^2^ (95% CI: 0.002, 0.018) and 0.012 g/cm^2^ (95% CI: 0.002, 0.022), respectively. Finally, women ≥ 45 years who changed from 0 to > 60 min/day of naps showed an increase in subtotal body, total hip, and lumbar spine increased by 0.011 g/cm^2^ (95% CI: 0.0007, 0.022), 0.012 (95% CI: 0.0004, 0.024), and 0.027 (95% CI: 0.009, 0.045), respectively (Fig. 1 and supplementary Table 4).

In addition, we conducted sensitivity analyses using alternative age cutoffs (< 47/ ≥ 47 and < 51/ ≥ 51) to women. These analyses were explored to assess the robustness of our findings. The results demonstrated a similar direction of association as observed when categorizing women as < 45/ ≥ 45 years, which served as a proxy for postmenopausal status (data not shown).

Due to the questionnaire being designed to record nap times if participants take them and the absence of an option for participants to indicate they do not take naps, potentially introducing misclassification bias, we conducted a sensitivity analysis by excluding these participants. The analysis generally maintained the same direction of results, though some lost statistical significance. Future research should incorporate options to capture participants’ nap habits better.

## Discussion

As the first study longitudinal to investigate the association between sleep and nap duration and BMD in a Mexican population, our findings indicate that increased sleep and nap duration in women are associated with gains in BMD at multiple body sites. These associations were not observed in men. Prior research on sleep, nap duration, and BMD has yielded mixed results, possibly due to variations in study design, sex stratification, variations in sleep and nap duration categories, diverse populations, measurement methods, and potential confounding factors[15–18, 41].

In agreement with our results, a cross-sectional study of 11,084 postmenopausal women found that those who slept ≤ 5 h per night had lower BMD and higher odds of osteoporosis (OR 1.63, 95% CI 1.15, 2.31), compared with those who slept 7 h per night[17]. Similarly, another study with 602 women (18–80 years) found that women with short sleep duration (≤ 5 h) had lower total and regional BMD compared to those sleeping 8 h daily[15].

Contrastingly, a cross-sectional study involving 8,688 participants reported higher risks of osteoporosis in postmenopausal women with longer sleep duration and daily napping, a trend not observed in men or premenopausal women. The risk was 57% higher for postmenopausal women sleeping 10 h/day or longer, compared to those sleeping 8–9 h/day, and 65% higher for daytime naps longer than 60 min/day, compared to no naps. [18]. A similar association was evaluated in a study of 6,510 pre- and post-menopausal women assessing sleep patterns and calcaneal BMD. Postmenopausal women with noontime naps (> 60 min vs. no nap) had a higher risk of BMD loss (OR: 1.37, 95% CI 1.06, 1.76). However, these associations were not found in premenopausal women[42]. It’s important to note that these associations are limited by their study design, making it challenging to establish a clear cause-effect relationship.

These divergent results underscore the complexity of the relationship between sleep duration, napping, and bone health, possibly influenced by various factors such as study design and population characteristics.

Although limited longitudinal research exists on the link between sleep duration and BMD, notable studies have emerged. One study with Indian postmenopausal women found a significant decline in BMD percentage in those with suboptimal sleep over two years[43]. Our study complements these findings, suggesting a potential association between sleep duration and BMD, while also highlighting the need for further investigation into napping.

In women aged ≥ 45 years, increased sleep and nap duration were associated with gains in BMD at multiple sites. Sleep has an essential role in health throughout every person life, and involves many biological and physiological processes, such as estrogen, which directly affects bone health, typically declines around age 45 during perimenopause[44]. A study conducted by Lin Jin et al. found varying associations between sleep quality and BMD across different menopausal stages, with significant links observed in premenopausal and early postmenopausal groups but not in late menopause in middle-aged women[45]. Sleep disturbances have been reported during menopause[46]. Notably, significant associations between sleep duration and naps were found only in postmenopausal women, not in men or premenopausal women[15, 18, 20]. This observation may be partly explained by the fact that men typically have greater bone mass, size, and a shorter lifespan without male equivalent of menopause, while BMD loss in women typically coincides with the onset of menopause[18, 43].

Limited evidence exists regarding the sleep-BMD association in men. Our results align with other studies showing no link between sleep duration and hip or lumbar spine BMD in older men[18, 47, 48]. Specker et al., used novel markers (other than BMD), which indicated that sleep deprivation in women was associated with lower cortical volumetric BMD, and sleep-deprived men had a lower torsional bending strength when compared with sleep-adequate counterparts[48]. Our findings emphasize the importance of considering gender, menopausal status, and novel markers in investigating the sleep-BMD relationship. The results add to our understanding of the complex interplay between sleep, hormonal changes, and bone health, but further research is needed to fully elucidate these relationships.

Sleep impacts various physiological processes, including hormone secretion related to bone metabolism and the sympathetic nervous system. Nevertheless, further research is needed to clarify these mechanisms. The effects of sleep on bone resorption and formation remain unclear, but an imbalance in these processes can increase fracture risk. Reduced sleep duration impacts growth hormone secretion and can lead to bone loss. Bone turnover markers peak during the early morning hours[49, 50], and animal studies have demonstrated that chronic short sleep duration can negatively affect bone metabolism, reducing BMD and altering microarchitecture[51, 52].

The observation that increased napping may serve as a surrogate for poor sleep patterns or an inability to stay awake adds an intriguing layer to the interpretation of our findings. While our study focuses on the association between changes in sleep and nap duration with bone mineral density (BMD), it is crucial to acknowledge the broader implications of extended napping habits. We observed interesting patterns regarding nap duration across different categories of nighttime sleep duration. In individuals with very short nighttime sleep (< 4 h/day), the median nap duration was 57.5 min (interquartile range: 16–77.1 min). Contrastingly, those in the short nighttime sleep group (5–6 h/day and 7–8 h/day) exhibited a notably shorter median nap duration of 7.5 min (interquartile range: 0–31.8 min). Meanwhile, individuals with long nighttime sleep durations (> 9 h/day) had a median nap duration of 25.7 min (interquartile range: 0–64.3 min). These findings shed light on the variation in nap duration across different nighttime sleep durations. Extended napping, when not attributable to planned rest or cultural practices, could indeed reflect underlying issues related to sleep quality or daytime sleepiness. Poor sleep patterns have been consistently linked to a myriad of adverse health outcomes[2–4]. Additionally, excessive daytime sleepiness may impact overall functioning and has been associated with impaired cognitive performance and a higher risk of accidents[1, 53]. Our findings emphasize the importance of not only considering sleep duration but also addressing the quality of sleep and daytime alertness. Future research could explore the reasons behind increased napping, whether it signifies inadequate nighttime sleep, sleep disorders, or other health-related factors.

This study represents the first longitudinal investigation of sleep and nap duration’s association with BMD changes in a Mexican population. The study’s strengths include its longitudinal design, which allowed us to assess changes in sleep and napping habits and account for confounding variables. Additionally, BMD was measured using DXA, considered the gold standard for assessing bone status. Our results also reflect sleep duration patterns similar to those reported in Mexico[22, 23]. Limitations include the focus on daily sleep and nap duration in the self-administered questionnaire, without assessing sleep quality. Self-reported sleep duration may include time spent in bed before falling asleep or nighttime awakenings. Moreover, BMD could not be categorized by t-scores due to limited changes in BMD categories within the study population. Information regarding medication use affecting sleep or napping was unavailable. Although not all associations reached statistical significance after adjusting for multiple comparisons, the study’s overall conclusions remain robust. Additionally, the statistical power for men was notably lower than for women, limiting the ability to detect significant associations in this subgroup. Given the sample size limitations and the number of comparisons conducted, larger studies with increased statistical power are warranted to validate and further explore these associations. An important limitation of our study is the lack of specific evaluation of degenerative changes in the spinal column. Although we have considered several factors that could affect bone mineral density (BMD), such as age, body mass index, and physical activity, we have not directly assessed degenerative changes in the spinal column. It is possible that the increases observed in lumbar spine BMD in our longitudinal analysis are related to the accumulation of degenerative changes rather than a real increase in BMD. We acknowledge that this is a significant limitation of our study and that direct evaluation of degenerative changes in future research could provide a more comprehensive understanding of our results.

## Conclusions

Our results suggest that an increase in sleep and nap duration are associated with gains in BMD at different skeletal sites in women. These findings not only underscore the significance of considering sleep as a potential factor for promoting optimal bone health but also support the importance of adhering to the established sleep recommendations. Further research is warranted to fully elucidate the causal mechanisms and develop specific sleep guidelines that can effectively enhance bone health.

### Supplementary Information

Below is the link to the electronic supplementary material.

Supplementary file1 (DOCX 34 KB)
