# Supplementary material for: Association Between Changes in Sleep, Nap Duration and Bone Mineral Density in Mexican Adults
Source: Calcif Tissue Int. 2024 May 17;115(1):31–40. doi: 10.1007/s00223-024-01224-1 (PMC11153263; doi:10.1007/s00223-024-01224-1)
Supplement: Supplementary file 1 — Supplementary file1 (DOCX 34 KB) [file 223_2024_1224_MOESM1_ESM.docx]

**Supplementary Table 1** Cross-sectional association of hours of sleep, nap duration and BMD in women by age groups

|  | **<45 years (n=527)** | **>45 years (n=593)** |
| --- | --- | --- |
|  | **β (% 95 CI)** | **β (% 95 CI)** |
|  | **Subtotal body BMD, g/cm^2^** | |
| Sleep duration^a^, hours/day | -0.002(-0.008,0.003) | 0.004(-0.0007,0.009) |
| < 7 hours/day^a^ | Ref. | Ref. |
| > 7 hours/day^a^ | 0.003(-0.019,0.017) | 0.002(-0.011,0.015) |
| <4h/day^a^ | Ref. | Ref. |
| 5-6h/day^a^ | -0.042(-0.089,0.006) | 0.036(-0.007,0.080) |
| 7-8 h/day^a^ | -0.035(-0.082,0.011) | 0.036(-0.006,0.079) |
| >9h/day | -0.040(-0.093,0.013) | 0.035(-0.013,0.083) |
| Napping^b^, min/day | -0.00001(-0.00005,0.00002) | 0.0000006(-0.00003,0.00004) |
| 0 min/day^b^ | Ref. | Ref. |
| < 30 min^b^ | -0.007(-0.021,0.008) | 0.0007(-0.014,0.015) |
| 30-60 min^b^ | -0.007(-0.027,0.012) | -0.009(-0.027,0.010) |
| >60 min^b^ | -0.003(-0.022,0.016) | -0.005(-0.022,0.012) |
| Sleep duration+ napping, hours/day | -0.002(-0.007,0.003) | 0.003(-0.001,0.008) |
|  | **Total hip BMD, g/cm^2^** | |
| Sleep duration^a^, hours/day | 0.00002(-0.009,0.009) | **0.010(0.003,0.017)**** |
| < 7 hours/day^a^ | Ref. | Ref. |
| > 7 hours/day^a^ | 0.006(-0.016,0.028) | 0.015(-0.005,0.034) |
| <4h/day^a^ | Ref | Ref. |
| 5-6h/day^a^ | -0.024(-0.105,0.057) | **0.073(0.006,0.140)*** |
| 7-8 h/day^a^ | -0.016(-0.096,0.063) | **0.083(0.017,0.149)*** |
| >9h/day | -0.012(-0.103,0.078) | **0.080(0.005,0.0154)*** |
| Napping^b^, min/day | -0.00003(-0.0001,0.00003) | 0.000002(-0.00005,0.00005) |
| 0 min/day^b^ | Ref. | Ref. |
| < 30 min^b^ | -0.010(-0.034,0.014) | -0.0005(-0.023,0.022) |
| 30-60 min^b^ | -0.018(-0.051,0.015) | -0.009(-0.039,0.020) |
| >60 min^b^ | -0.007(-0.039,0.025) | -0.017(-0.043,0.009) |
| Sleep duration+ napping, hours/day | -0.002(-0.010,0.007) | **0.009(0.002,0.015)*** |
|  | **Lumbar spine BMD, g/cm^2^** | |
| Sleep duration^a^, hours/day | -0.0009(-0.012,0.010) | 0.009(-0.004,0.019) |
| < 7 hours/day^a^ | Ref. | Ref. |
| > 7 hours/day^a^ | 0.008(-0.018,0.035) | 0.016(-0.010,0.043) |
| <4h/day^a^ | Ref. | Ref. |
| 5-6h/day^a^ | -0.042(-0.138,0.054) | 0.076(-0.013,0.166) |
| 7-8 h/day^a^ | -0.030(-0.125,0.064) | 0.087(-0.0006,0.175) |
| >9h/day | -0.050(-0.157,0.058) | 0.089(-0.009,0.188) |
| Napping^b^, min/day | -0.00004(-0.0001,0.00004) | -0.00004(-0.0001,0.00003) |
| 0 min/day^b^ | Ref. | Ref. |
| < 30 min^b^ | -0.012(-0.040,0.017) | 0.009(-0.021,0.039) |
| 30-60 min^b^ | -0.019(-0.058,0.021) | 0.007(-0.032,0.045) |
| >60 min^b^ | -0.001(-0.040,0.037) | -0.009(-0.044,0.026) |
| Sleep duration+ napping, hours/day | -0.002(-0.012,0.008) | 0.006(-0.003,0.015) |
| Model adjusted for age, body mass index, smoking status (never, former smoker), DII, PA, calcium supplements, calcium intake, diabetes, and hormone replacement therapy. ^a^ This model includes a napping adjustment ^b^ This model includes a sleep duration adjustment * p<0.05, ** p<0.01, *** p<0.001 | | |

**Supplementary Table 2** Cross-sectional association of hours of sleep, nap duration and low-BMD in women by age groups

|  | **<45 years (n=527)** | **>45 years (n=593)** |
| --- | --- | --- |
|  | **OR (% 95 CI)** | **OR (% 95 CI)** |
|  | **Low Total hip BMD, g/cm^2^** | |
| Sleep duration^a^, hours/day | 1.15(0.85-1.55) | **0.84(0.71-0.99)*** |
| < 7 hours/day^a^ | Ref. | Ref. |
| > 7 hours/day^a^ | 1.25(0.62-2.50) | 0.64(0.39-1.05) |
| <4h/day^a^ | Ref. | Ref. |
| 5-6h/day^a^ | - | **0.10(0.02-0.47)**** |
| 7-8 h/day^a^ | 2.77(0.31-24.76) | **0.09(0.02-0.39)**** |
| >9h/day | 3.41(0.41-28.02) | **0.11(0.02-0.61)*** |
| Napping^b^, min/day | 1.00(0.99-1.00) | 1.00(0.99-1.00) |
| 0 min/day^b^ | Ref. | Ref. |
| < 30 min^b^ | 0.93(0.45-1.90) | 1.21(0.74-1.99) |
| 30-60 min^b^ | 1.56(0.62-3.94) | 1.36(0.72-2.55) |
| >60 min^b^ | 1.46(0.56-3.80) | 1.37(0.78-2.43) |
| Sleep duration+ napping, hours/day | 1.17(0.87-1.56) | **0.84(0.73-0.98)*** |
|  | **Low Lumbar spine BMD, g/cm^2^** | |
| Sleep duration^a^, hours/day | 0.98(0.80-1.20) | 0.90(0.77-1.05) |
| < 7 hours/day^a^ | Ref. | Ref. |
| > 7 hours/day^a^ | 0.99(0.62-1.62) | 0.88(0.59-1.31) |
| <4h/day^a^ | Ref. | Ref. |
| 5-6h/day^a^ | 1.40(0.16-12.56) | 0.35(0.07-1.88) |
| 7-8 h/day^a^ | 1.37(0.16-11.95) | 0.33(0.06-1.75) |
| >9h/day | 1.55(0.15-16.11) | 0.27(0.04,1.66) |
| Napping^b^, min/day | 1.00(0.99-1.00) | 1.00(0.99-1.00) |
| 0 min/day^b^ | Ref. | Ref. |
| < 30 min^b^ | 0.89(0.53-1.48) | 0.93(0.59-1.45) |
| 30-60 min^b^ | 0.83(0.39-1.73) | 0.92(0.52-1.64) |
| >60 min^b^ | 1.35(0.69-2.64) | 1.14(0.66-1.98) |
| Sleep duration+ napping, hours/day | 1.05(0.90-1.23) | 0.95(0.84-1.08) |
| Model adjusted for age, body mass index, smoking status (never, former smoker), DII, PA, calcium supplements, calcium intake, diabetes, and hormone replacement therapy. ^a^ This model includes a napping adjustment ^b^ This model includes a sleep duration adjustment * p<0.05, ** p<0.01, *** p<0.001. Low-BMD as a T-score below -1 at the total hip, and lumbar spine. | | |

**Supplementary Table 3** BMD change according to Changes in sleep and nap duration between baseline and follow-up by sex

|  | **Males (n=341)** | **Females (n=996)** |
| --- | --- | --- |
|  | **β (% 95 CI)** | **β (% 95 CI)** |
| **Crude model** | **Subtotal body BMD, g/cm^2^** | |
| Sleep duration, hours/day | 0.0007(-0.002,0.003) | **0.002(0.00006,0.004)*** |
| Change < 7 hours/day to > 7 hours/day | 0.002(-0.006,0.009) | 0.003(-0.003,0.008) |
| Change from <4h/day to 5-6h/day | 0.005(-0.012,0.023) | 0.011(-0.001,0.023) |
| Change from <4h/day to 7-8 h/day | 0.006(-0.011,0.023) | **0.012(0.0003,0.024)*** |
| Change from <4h/day to >9h/day | 0.014(-0.010,0.038) | 0.014(-0.0009,0.029) |
| Napping, min/day | 0.003(-0.001,0.008) | 0.002(-0.0003,0.005) |
| Change 0 min/day to < 30 min | 0.0009(-0.008,0.010) | **0.006(0.004,0.011)*** |
| Change 0 min/day to 30-60 min | **0.014(0.002,0.026)*** | 0.003(-0.004,0.011) |
| Change 0 min/day to>60 min | 0.008(-0.003,0.019) | **0.011(0.004,0.018)*** |
| Sleep duration+ napping, hours/day | 0.001(-0.0008,0.004) | **0.002(0.0005,0.003)**** |
| **Adjusted model** | **Subtotal body BMD, g/cm^2^** | |
| Sleep duration^a^, hours/day | 0.0007(-0.002,0.003) | 0.002(-0.00003,0.003) |
| Change < 7 hours/day to > 7 hours/day ^a^ | 0.001(-0.007,0.009) | 0.003(-0.003,0.008) |
| Change from <4h/day to 5-6h/day ^a^ | 0.006(-0.011,0.024) | 0.010(-0.0018,0.022) |
| Change from <4h/day to 7-8 h/day ^a^ | 0.006(-0.011,0.023) | 0.011(-0.0004,0.023) |
| Change from <4h/day to >9h/day ^a^ | 0.015(-0.010,0.039) | 0.013(-0.001,0.028) |
| Napping^b^, hours/day | 0.003(-0.002,0.008) | 0.001(-0.001,0.004) |
| Change 0 min/day to < 30 min ^b^ | -0.0002(-0.010,0.009) | **0.006(0.0004,0.011)*** |
| Change 0 min/day to 30-60 min ^b^ | **0.012(0.00006,0.024)*** | 0.004(-0.003,0.011) |
| Change 0 min/day to >60 min ^b^ | 0.007(-0.005,0.018) | **0.009(0.002,0.016)*** |
| Sleep duration+ napping, hours/day | 0.001(-0.001,0.003) | **0.002(0.0001,0.003)*** |
| **Crude model** | **Total hip BMD, g/cm^2^** | |
| Sleep duration, hours/day | 0.003(-0.00002,0.006) | **0.012(0.005,0.019)**** |
| Change < 7 hours/day to > 7 hours/day | 0.006(-0.003,0.015) | **0.012(0.005,0.019)***** |
| Change from <4h/day to 5-6h/day | 0.015(-0.007,0.037) | **0.016(0.0008,0.031)*** |
| Change from <4h/day to 7-8 h/day | 0.018(-0.003,0.040) | **0.026(0.011,0.041)**** |
| Change from <4h/day to >9h/day | 0.028(-0.002,0.058) | **0.022(0.003,0.041)*** |
| Napping, min/day | 0.005(-0.001,0.010) | 0.002(-0.001,0.006) |
| Change 0 min/day to < 30 min | 0.002(-0.010,0.013) | 0.002(-0.005,0.009) |
| Change 0 min/day to 30-60 min | 0.008(-0.007,0.022) | 0.004(-0.006,0.013) |
| Change 0 min/day to>60 min | **0.017(0.0003,0.030)*** | **0.014(0.005,0.023)**** |
| Sleep duration+ napping, hours/day | **0.004(0.0008,0.006)*** | **0.003(0.0010,0.005)**** |
| **Adjusted model** | **Total hip BMD, g/cm^2^** | |
| Sleep duration^a^, hours/day | 0.005(-0.004,0.014) | **0.011(0.004,0.017)**** |
| Change < 7 hours/day to > 7 hours/day ^a^ | 0.007(-0.002,0.016) | **0.011(0.004,0.017)**** |
| Change from <4h/day to 5-6h/day ^a^ | 0.011(-0.0109,0.032) | **0.016(0.0007,0.031)*** |
| Change from <4h/day to 7-8 h/day ^a^ | 0.014(-0.007,0.035) | **0.025(0.010,0.039)**** |
| Change from <4h/day to >9h/day ^a^ | 0.026(-0.004,0.055) | **0.022(0.003,0.040)*** |
| Napping^b^, hours/day | 0.003(-0.003,0.008) | 0.001(-0.002,0.005) |
| Change 0 min/day to < 30 min ^b^ | -0.001(-0.012,0.010) | 0.002(-0.004,0.009) |
| Change 0 min/day to 30-60 min ^b^ | 0.003(-0.011,0.018) | 0.004(-0.005,0.013) |
| Change 0 min/day to >60 min ^b^ | 0.013(-0.0007,0.026) | **0.012(0.003,0.020)**** |
| Sleep duration+ napping, hours/day | 0.002(-0.0002,0.005) | **0.002(0.0005,0.004)*** |
| **Crude model** | **Lumbar spine BMD, g/cm^2^** | |
| Sleep duration, hours/day | 0.003(-0.0009,0.007) | **0.005(0.002,0.008)**** |
| Change < 7 hours/day to > 7 hours/day | 0.003(-0.008,0.015) | **0.017(0.006,0.027)**** |
| Change from <4h/day to 5-6h/day | 0.023(-0.005,0.050) | 0.0098(-0.016,0.033) |
| Change from <4h/day to 7-8 h/day | 0.023(-0.004,0.005) | 0.025(0.005,0.048) |
| Change from <4h/day to >9h/day | 0.031(-0.007,0.069) | 0.026(-0.005,0.056) |
| Napping, min/day | -0.006(-0.013,0.001) | **0.007(0.002,0.013)**** |
| Change 0 min/day to < 30 min | 0.0001(-0.014,0.014) | 0.007(-0.004,0.017) |
| Change 0 min/day to 30-60 min | 0.008(-0.010,0.026) | 0.008(-0.006,0.023) |
| Change 0 min/day to>60 min | -0.011(-0.029,0.0006) | **0.021(0.007,0.036)**** |
| Sleep duration+ napping, hours/day | 0.001(-0.002,0.004) | **0.005(0.003,0.008)***** |
| **Adjusted model** | **Lumbar spine BMD, g/cm^2^** | |
| Sleep duration^a^, hours/day | 0.003(-0.001,0.007) | **0.005(0.001,0.008)**** |
| Change < 7 hours/day to > 7 hours/day ^a^ | 0.002(-0.009,0.014) | **0.017(0.006,0.027)**** |
| Change from <4h/day to 5-6h/day ^a^ | 0.018(-0.010,0.046) | 0.005(-0.019,0.030) |
| Change from <4h/day to 7-8 h/day ^a^ | 0.018(-0.010,0.046) | 0.021(-0.003,0.045) |
| Change from <4h/day to >9h/day ^a^ | 0.031(-0.008,0.069) | 0.025(-0.006,0.055) |
| Napping^b^, hours/day | -0.006(-0.013,0.002) | **0.007(0.001,0.012)*** |
| Change 0 min/day to < 30 min ^b^ | -0.002(-0.016,0.013) | 0.007(-0.003,0.017) |
| Change 0 min/day to 30-60 min ^b^ | 0.006(-0.012,0.024) | 0.011(-0.003,0.025) |
| Change 0 min/day to >60 min ^b^ | -0.012(-0.030,0.005) | **0.021(0.007,0.035)**** |
| Sleep duration+ napping, hours/day | 0.0009(-0.003,0.004) | **0.005(0.002,0.008)***** |
| Model adjusted for body mass index, smoking status (never, former smoker), DII, PA, calcium supplements, calcium intake, diabetes, and hormone replacement therapy. ^a^ This model includes a napping adjustment ^b^ This model includes a sleep duration adjustment. * p<0.05, ** p<0.01, *** p<0.001 | | |

**Supplementary Table 4** BMD change according to Changes in sleep and nap duration between baseline and follow-up in women stratified by age

|  | **<45 (n=450)** | **>45 (n=546)** |
| --- | --- | --- |
|  | **β (% 95 CI)** | **β (% 95 CI)** |
| **Adjusted model** | **Subtotal body BMD, g/cm^2^** | |
| Sleep duration^a^, hours/day | -0.0004(-0.003,0.002) | **0.003(0.0009,0.005)**** |
| Change < 7 hours/day to > 7 hours/day^a^ | -0.006(-0.013,0.00004) | **0.013(0.005,0.021)**** |
| Napping^b^, hours/day | -0.00001(-0.00007,0.00005) | 0.00004(-0.00002,0.0001) |
| Change 0 min/day to < 30 min ^b^ | 0.005(-0.002,0.011) | 0.004(-0.004,0.011) |
| Change 0 min/day to 30-60 min ^b^ | -0.002(-0.012,0.007) | 0.009(-0.001,0.019) |
| Change 0 min/day to>60 min ^b^ | 0.004(-0.007,0.014) | **0.011(0.001,0.020)*** |
| Sleep duration+ napping, hours/day | -0.0005(-0.003,0.002) | **0.003(0.001,0.005)**** |
| **Adjusted model** | **Total hip BMD, g/cm^2^** | |
| Sleep duration^a^, hours/day | **0.003(0.0001,0.006)*** | 0.003(-0.0004,0.005) |
| Change < 7 hours/day to > 7 hours/day^a^ | **0.010(0.002,0.018)*** | **0.012(0.002,0.022)*** |
| Napping^b^, hours/day | 0.000005(-0.00007,0.00008) | 0.00003(-0.00005,0.0001) |
| Change 0 min/day to < 30 min ^b^ | -0.001(-0.009,0.007) | 0.005(-0.005,0.015) |
| Change 0 min/day to 30-60 min ^b^ | -0.003(-0.014,0.009) | 0.010(-0.003,0.023) |
| Change 0 min/day to>60 min ^b^ | 0.010(-0.003,0.023) | **0.012(0.0004,0.024)*** |
| Sleep duration+ napping, hours/day | 0.002(-0.0001,0.005) | 0.002(-0.002,0.005) |
| **Adjusted model** | **Lumbar spine BMD, g/cm^2^** | |
| Sleep duration^a^, hours/day | **0.006(0.0003,0.012)*** | **0.004(0.00002,0.009)*** |
| Change < 7 hours/day to > 7 hours/day^a^ | 0.011(-0.004,0.026) | **0.024(0.009,0.039)**** |
| Napping^b^, hours/day | 0.00006(-0.00008,0.0002) | **0.0001(0.00003,0.0003)*** |
| Change 0 min/day to < 30 min ^b^ | -0.003(-0.018,0.012) | **0.015(0.0001,0.029)*** |
| Change 0 min/day to 30-60 min ^b^ | -0.005(-0.027,0.016) | **0.024(0.005,0.043)*** |
| Change 0 min/day to>60 min ^b^ | 0.009(-0.014,0.033) | **0.027(0.009,0.045)**** |
| Sleep duration+ napping, hours/day | **0.005(0.0006,0.010)*** | **0.005(0.002,0.009)**** |
| Model adjusted for body mass index, smoking status (never, former smoker), DII, PA, calcium supplements, calcium intake, diabetes, and hormone replacement therapy. ^a^ This model includes a napping adjustment ^b^ This model includes a sleep duration adjustment. | | |
| * p<0.05, ** p<0.01, *** p<0.001 |  |  |
